# Epidemiology and Economic Impact of Celiac Disease in the South Vesuvian Area of Naples: A Survey

**DOI:** 10.3389/fpubh.2013.00018

**Published:** 2013-06-13

**Authors:** Maurizio Capuozzo, Alessandro Ottaiano, Eduardo Nava, Stefania Cascone, Claudia Cinque, Rosario V. Iaffaioli, Corinne Scognamiglio, Emilia Palumbo, Maria Capuozzo

**Affiliations:** ^1^Department of Pharmacy, Local Sanitary Agency (LSA) Naples 3 SouthNaples, Italy; ^2^Department of Colorectal Oncology, National Cancer Institute, “G. Pascale” FoundationNaples, Italy; ^3^Local Sanitary Agency (LSA) Naples 1 CenterNaples, Italy; ^4^Department of Pharmacology, University “Federico II”Naples, Italy

## Introduction

Celiac disease is a chronic digestive disorder of genetic origin in which damage to the lining of the small intestine, caused by an immunological reaction to gluten, leads to the malabsorption of minerals and nutrients (1). Signs and symptoms of celiac disease may vary among individuals depending on the degree of malabsorption: they range from absent, few or mild, to many or severe. There is no specific cure for celiac disease; the main treatment is a gluten-free diet (2).

The social impact of celiac disease is very high and its estimated prevalence in Italy is about 1.5%. The Italian law no.123 (July 4, 2005) recognizes the celiac disease as “social disease” and ensures the free distribution of gluten-free aliments. However, there is no national legislation regulating the delivery modalities of gluten-free foods for patients with celiac disease; there are only some regional laws or even internal corporate regulations that establish the paths of delivery of gluten-free foods.

In the Local Sanitary Agency (LSA) Naples 3 South Italy (i.e., LSA, NA 3 South), patients with celiac disease are taken into care by the Units of Basic Medicine of their Health District (a subunit of the LSA); subsequently, the pharmaceutical service emits a booklet composed of 12 coupons corresponding to the 12 months of the year. The value in euro of each coupon varies according to age and gender of the patient as reported in a table of a specific Ministerial Decree (May 4, 2006); it determines exactly the expenditure cut-off for the delivery of gluten-free products. Patients can buy gluten-free foods using these coupons in any territorial pharmacy.

Given the significant social impact of celiac disease and the increasing number of cases, we conducted a survey with the aim (i) to show the updated prevalence of the disease in a defined population and (ii) to evaluate the economic impact.

## Methods

We studied the population of the South Vesuvian area of Naples (Italy). It comprises eight Sanitary Districts and a total resident population of 558.939 as reported on January 2012 according to ISTAT (Istituto nazionale di STATistica, National Institute of Statistics); 271.039 were males (M) and 287.900 females (F). Each pharmaceutical service has a database (Report Data Celiac disease – RDC) in an electronic format which converges through a central coordination service into a single regional database. The RDC stratifies the population in groups by age (0–1, 2–4, 5–10, 11–18, 19–65, >65 years), and gender (M vs. F). The number of celiac patients and the related expenditure of the LSA (ASL NA 3 South) are reported for each class. The final database is then transmitted to the Pharmaceutical Sector of the Campania Region (Italy). The value in euro of each coupon is calculated according to a table of the Ministerial Decree (May 4, 2006): for the age group between 6 months and 1 year the value of each coupon is € 45 (M = F); 1–3.5 years, the value is € 62 (M = F); >3.5–10 years, the value is € 94 (M = F); for 10 years or older, the value of each coupon corresponds to € 140 for males and € 99 for females. Additionally, in the District of Herculaneum, considered as “pilot district,” a short interview with a pre-printed form was administered to patients with celiac disease (to parents in patients younger than 18 years) in order to assess the degree of satisfaction of their food needs.

## Results

In the observed population (see Methods), we found 1453 celiac subjects, 497 were male (M) and 956 female (F). The prevalence of celiac disease was 2.6/1000, with a prevalence of 1.8/1000 in male and 3.3/1000 in female. The male to female ratios (M/F) in specific age classes were: in the range of 0–1 years, 32.0% M and 68.0% F (0.47); in the range 2–4 years, 33.3% M and 66.7% F (0.49); in the range of 5–10 years, 37.1% M and 62.9% F (0.58); in the range 11–18 years, 36.0% M and 64.0% F (0.56); in the range of 19–65 years, 28.8% M and 71.2% F (0.40); in patients >65 years, 37.5% M and 62.5% F (0.6). The costs were calculated according to the above mentioned criteria (see Methods); they amounted to € 1620 for patients in the first class of age (6 months–1 year), € 33.480 for patients in the second class (1–3.5 years), € 315.840 for patients in the third class (>3.5–10 years), and € 1.524.444 for patients in the fourth class (>10 year). The costs according to gender were: € 641.760 for male and € 882.684 for female. The total health expenditure was € 1.875.384.

In the district of Herculaneum, considered as a “pilot district,” 166 patients were interviewed (namely, 11.4% of total patients); 58 were M and 108 F. The interviews were anonymous and consisted on three brief questions: “How old are you?,” “What is your gender?,” and “Is the value of the coupon enough for you?” The compliance to the questionnaire was very high. We found that 52.0% of patients believes that the budget is sufficient, 45.0% insufficient and only 3.0% did not respond to the questionnaire.

## Discussion

Celiac disease is a medical condition with high social impact. The disease is currently underestimated; however, a significant increase of affected patients has been observed over the last years with the improvement of diagnostic tools (i.e., laboratory techniques) particularly in the age range of 0–1 year (3). In the other age classes it is registered an exponential increase of cases. For all classes of age, the M/F ratio is about 1/2, with a higher prevalence in women. Adolescence is a critical phase of risk for the development of the intolerance to gluten. However, about 50% of cases are diagnosed in the adult stage (3). In the coming years, we expect an increasing number of celiac patients over the age of 65 years, with strong implications in health care and economy.

According to the guidelines of the National Institute for Clinical Excellence in the UK (4), undiagnosed celiac disease is associated with a high number of long-term complications, including osteoporosis and some malignant tumors. This suggests that significant savings may originate from changes in diet, with the use of gluten-free products. In fact, the high economic impact of celiac disease cannot be ignored. Our survey conducted in a population of 558.939 inhabitants of a single LSA of an Italian region shows a high health expenditure (€ 1.875.384); these costs will augment with the increasing prevalence of the disease. Data emerging from our interviews suggest that resources allocated monthly to patients are quite adequate. However, prevention of the disease is the best target, not only for the obvious benefits for patients, but also for a strong reduction of sanitary costs.
